# Diffuse Myocardial Fibrosis Reduces Electrocardiographic Voltage Measures of Left Ventricular Hypertrophy Independent of Left Ventricular Mass

**DOI:** 10.1161/JAHA.116.003795

**Published:** 2017-01-22

**Authors:** Maren Maanja, Björn Wieslander, Todd T. Schlegel, Ljuba Bacharova, Hussein Abu Daya, Yaron Fridman, Timothy C. Wong, Erik B. Schelbert, Martin Ugander

**Affiliations:** ^1^ Department of Clinical Physiology Karolinska Institutet, and Karolinska University Hospital Stockholm Sweden; ^2^ Department of Medicine University of Pittsburgh Medical Center Pittsburgh PA; ^3^ Nicollier‐Schlegel SARL Trélex Switzerland; ^4^ International Laser Center Bratislava Slovak Republic; ^5^ Institute of Pathophysiology Medical School Comenius University Bratislava Slovak Republic

**Keywords:** electrocardiography, extracellular matrix, hypertrophy, left ventricular, magnetic resonance imaging, Electrocardiology (ECG), Magnetic Resonance Imaging (MRI), Hypertrophy

## Abstract

**Background:**

Myocardial fibrosis quantified by myocardial extracellular volume fraction (ECV) and left ventricular mass (LVM) index (LVMI) measured by cardiovascular magnetic resonance might represent independent and opposing contributors to ECG voltage measures of left ventricular hypertrophy (LVH). Diffuse myocardial fibrosis can occur in LVH and interfere with ECG voltage measures. This phenomenon could explain the decreased sensitivity of LVH detectable by ECG, a fundamental diagnostic tool in cardiology.

**Methods and Results:**

We identified 77 patients (median age, 53 [interquartile range, 26–60] years; 49% female) referred for contrast‐enhanced cardiovascular magnetic resonance with ECV measures and 12‐lead ECG. Exclusion criteria included clinical confounders that might influence ECG measures of LVH. We evaluated ECG voltage‐based LVH measures, including Sokolow‐Lyon index, Cornell voltage, 12‐lead voltage, and the vectorcardiogram spatial QRS voltage, with respect to LVMI and ECV. ECV and LVMI were not correlated (*R* ^2^=0.02; *P*=0.25). For all voltage‐related parameters, higher LVMI resulted in greater voltage (*r*=0.33–0.49; *P*<0.05 for all), whereas increased ECV resulted in lower voltage (*r*=−0.32 to −0.57; *P*<0.05 for all). When accounting for body fat, LV end‐diastolic volume, and mass‐to‐volume ratio, both LVMI (β=0.58, *P*=0.03) and ECV (β=−0.46, *P*<0.001) were independent predictors of QRS voltage (multivariate adjusted *R* ^2^=0.39; *P*<0.001).

**Conclusions:**

Myocardial mass and diffuse myocardial fibrosis have independent and opposing effects upon ECG voltage measures of LVH. Diffuse myocardial fibrosis quantified by ECV can obscure the ECG manifestations of increased LVM. This provides mechanistic insight, which can explain the limited sensitivity of the ECG for detecting increased LVM.

## Introduction

Diffuse myocardial fibrosis and increased left ventricular (LV) mass may represent independent and opposing contributors to ECG voltage measures of left ventricular hypertrophy (LVH). Decreased ECG voltage from myocardial fibrosis could explain the limited sensitivity of LVH detectable by ECG, a fundamental diagnostic parameter in cardiology. LVH is broadly defined as an increased left ventricular mass (LVM)^1^ and remains relevant because LVH usually indicates prognostically adverse myocardial remodeling. In addition to enlargement of cardiomyocytes, pathologically hypertrophied myocardium undergoes complex changes in the interstitium, including diffuse myocardial fibrosis, defined as a global abnormal accumulation of collagen in the extracellular matrix of the myocardium.^2^ The cardiomyocyte and interstitial compartments are thought to be regulated independently of one another.^3^ Previously, myocardial fibrosis was measured only by invasive myocardial biopsy. Novel extracellular volume fraction (ECV) measures with cardiac magnetic resonance (CMR) have now been repeatedly histologically validated for noninvasive quantification of diffuse myocardial fibrosis.^4^, ^5^, ^6^, ^7^, ^8^, ^9^ In summary, quantitative histomorphometry studies showed a strong correlation between myocardial collagen fraction and myocardial ECV fraction by CMR in patients with aortic stenosis (*R* ^2^=0.86; *P*<0.001)^4^ and (*R* ^2^=0.69; *P*<0.01).^6^ Another study showed a strong correlation (*r*=0.85; *P*=0.01) between endomyocardial biopsies and myocardial ECV fraction by CMR in patients with dilated cardiomyopathy.^7^

No data exist on the contributions of diffuse myocardial fibrosis to ECG changes in LVH despite the central diagnostic role that the ECG occupies in cardiology. Several criteria for LVH from both the standard 12‐lead ECG and the vectorcardiogram (VCG) have been used, including the Sokolow–Lyon index, Cornell voltage criteria, and others.^10^, ^11^ However, diagnostic performance of LVH by ECG varies greatly, with sensitivity typically close to 50% and specificity approaching 90%,^10^ presumably attributable to confounding by myocardial fibrosis or other conditions.

To evaluate the relationship between diffuse myocardial fibrosis and ECG voltage, we evaluated LVH‐associated changes in the ECG with respect to LVM indexed to body surface area (LVMI) and ECV, as well as body fat, end‐diastolic volume (EDV), and LVM/EDV^2/3^, a surrogate for mass‐to‐volume ratio.^12^ We studied carefully selected patients who had varying levels of LVM and ECV but who lacked any clinical or CMR‐related findings that might otherwise obviously influence ECG measures of LVH. The hypothesis of the study was that both ECV and LVMI are independent and opposing contributors to ECG voltage criteria for LVH.

## Methods

### Study Patients

In this cross‐sectional observational study, patients were identified from a prospectively acquired database of 1707 enrolled patients who had undergone a clinically indicated CMR scan at University of Pittsburgh Medical Center (UPMC; Pittsburgh, PA) between 2010 and 2014. Inclusion criteria were ECG with sinus rhythm with a heart rate <100 beats/min and an ECG acquired within 30 days of CMR. The study was approved by the UPMC Institutional Review Board, and all participants provided written informed consent. Exclusion criteria were ECG confounders such as previous myocardial infarction (MI) or nonischemic myocardial scar as determined by late gadolinium enhancement (LGE) CMR, treatment with digitalis, atrial fibrillation or flutter, bundle branch‐ or fascicular block, abundant premature ventricular contractions (bigeminy/trigeminy), abundant premature atrial contractions, QRS duration >110 ms, paced rhythm, LV end‐diastolic volume (EDV) index (EDVI) >120 g/m^2^, body mass index (BMI) <18 or >30 kg/m^2^, previous cardiac surgery, pathologic cardiac stress perfusion CMR, cardiac amyloidosis, severe valvular disease, and significant coronary artery stenosis by invasive angiography.

**Figure 1:**
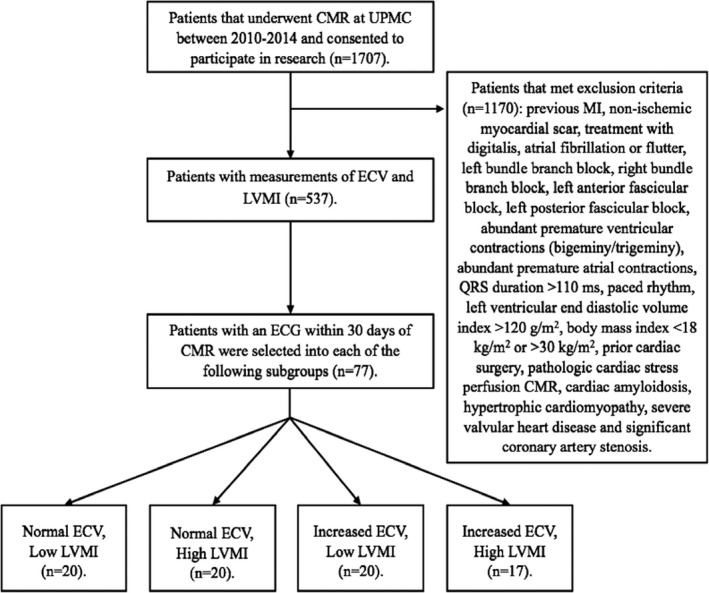
Flow chart of patient selection. The cutoff separating low from high left ventricular mass index (LVMI) was 55 g/m^2^, and the cutoff separating normal from increased myocardial extracellular volume fraction (ECV) was 28.5%. See Methods for details on cut off criteria and selection of patients into the 4 subgroups. CMR indicates cardiovascular magnetic resonance; ECV, extracellular volume; LMVI, left ventricular mass index; MI, myocardial infarction; UPMC, University of Pittsburgh Medical Center.

Based on an alpha of 0.05, power of 0.9, and a minimum detectable correlation coefficient (*r*) of 0.4, our power calculation^13^ resulted in a required sample size of n=62. In order to provide a margin of error, we set our target sample size at n=80 patients. Patients were divided into 4 groups according to the CMR parameters of LVMI and ECV, as shown in Figure 1, with a target sample size of n=20 in each group, with as equal contributions of both sexes as possible. Selection of subjects into groups was performed before ECG analysis in order to achieve a study population with a balanced and broad range of ECV and LVMI findings. A total of 77 patients were identified; 20 in 3 groups and 17 in the group with increased ECV and high LVMI, because of a shortage of eligible patients in that group who did not have ECG confounders.

Baseline data included age at CMR, sex, LVM, LVMI, ECV, EDV, EDVI, left ventricular ejection fraction (LVEF), BMI, body surface area (BSA), tobacco use, hypertension, diabetes mellitus, treatment with beta‐blockers, angiotensin‐converter enzyme inhibitors (ACEi), angiotensin receptor blockers (ARB), and diuretics as reflected in the medical record. Body fat was derived from BMI, as described by Deurenberg et al,^14^ according to the formula: adult body fat (%)=(1.20×BMI)+(0.23×Age)−(10.8×sex)−5.4, where sex is 1 for males and 0 for females.

### Subgroups

From a database of 1707 patients, 1170 met the exclusion criteria and 537 had data for both LVMI and myocardial ECV measurements, as shown in Table 1. We aimed to achieve a study population with both sexes equally represented, as well as with wide ranges of LVMI and ECV. Therefore, the target sample size was 10 subjects of each sex in the following subgroups: (1) normal ECV (defined as <28.5% based on normal values from volunteers at UPMC) and low LVMI (defined as ≤55 g/m^2^ based on the distribution of LVMI in the study population; see below); (2) normal ECV and high LVMI (>55 g/m^2^); (3) increased ECV (≥28.5%) and low LVMI; and (4) both increased ECV and high LMVI.

**Table 1:** Baseline Variables for the Different Subgroups

Characteristics	All	ECV Normal, LVMI Low	ECV Normal, LVMI High	ECV Increased, LVMI Low	ECV Increased, LVMI High	*P* Value
No., n	77	20	20	20	17	0.95
Age, y	53 (26–60)	48 (25–59)	47 (24–60)	54 (48–63)	54 (31–60)	0.65
Female sex	38 (49)	10 (50)	10 (50)	9 (45)	9 (53)	0.99
LVM, g	102 (85–126)	83 (80–90)	139 (124–156)	86 (75–95)	117 (102–138)	<0.001
LVMI, g/m^2^	55 (45–68)	45 (43–46)	75 (68–78)	46 (42–50)	64 (59–74)	<0.001
ECV, %	28.5±3.8	26.3±1.8	24.9±2.4	30.9±2.6	32.3±2.6	<0.001
EDV, mL	149.0±37.0	136.2±28.6	164.6±34.4	137.2±39.3	159.6±38.9	0.02
EDVI, mL/m^2^	79.3±17.1	72.6±14.3	86.2±12.7	71.7±18.0	87.9±17.5	0.001
LVEF, %	63 (57–67)	64 (59–68)	62 (57–67)	63 (58–69)	60 (52–66)	0.52
LVM/EDV^2/3^, g/mL^2/3^	3.9±0.9	3.3±0.5	4.8±0.8	3.4±0.5	4.2±0.7	<0.001
BMI, kg/m^2^	25.2 (22.8–28.1)	24.6 (22.7–27.7)	25.3 (23.6–28.1)	26.7 (24.8–28.2)	24.1 (21.6–27.9)	0.43
Body fat, %	30.1±7.2	28.9±8.4	29.8±9.2	31.7±4.9	29.8±4.9	0.63
BSA, m^2^	1.9±0.2	1.9±0.2	1.9±0.2	1.9±0.2	1.8±0.2	0.54
Hypertension	26 (34)	8 (40)	4 (20)	6 (30)	8 (47)	0.64
Diabetes mellitus	7 (9)	0 (0)	2 (10)	1 (5)	4 (24)	0.17
Smoking status						
Current smoker	8 (10)	0 (0)	3 (15)	2 (10)	3 (18)	0.39
Ex‐smoker	28 (36)	10 (50)	6 (30)	6 (30)	6 (35)	0.63
Medication						
Beta‐blockers	19 (25)	6 (30)	4 (20)	3 (15)	6 (35)	0.70
ACEi/ARB	18 (23)	3 (15)	7 (35)	3 (15)	5 (29)	0.49
Diuretics	29 (38)	6 (30)	9 (45)	7 (35)	7 (41)	0.88

Continuous data are given as mean±SD, median (interquartile range), or number (%). ACEi indicates angiotensin‐converting enzyme inhibitors; ARB, angiotensin II receptor blocker; BMI, body mass index; BSA, body surface area; CMR, cardiovascular magnetic resonance; ECV, extracellular volume fraction; EDV, end‐diastolic volume; EDVI, end‐diastolic volume index; LVEF, left ventricular ejection fraction; LVM, left ventricular mass; LVMI, left ventricular mass index.

There are several sources for LVMI reference ranges in the literature with varying limits.^15^, ^16^ We adopted a subgroup division strategy based on quartiles of LVMI. Specifically, we first calculated the quartile ranges of the 537 patients who met inclusion criteria and that had data on LVMI and ECV available. Next, we enrolled subjects for the high‐LVMI subgroups from the fourth quartile and downward. Because this approach did not identify 20 patients for each subgroup, we further included patients with the highest LVMI from the third quartile. In a corresponding fashion, we enrolled subjects for the low‐LVMI subgroups starting from the bottom of the second quartile and upward. To meet the sample size, we further included patients with the highest LVMI from the first quartile as well as patients with the lowest LVMI from the third quartile. Finally, this approach identified 55 g/m^2^ as the cutoff between high and low LVMI in the population.

### CMR Acquisition and Analysis

#### Quantification of volumes and dimensions

CMR images were acquired with a 1.5 Tesla scanner (Magnetom Espree; Siemens Medical Solutions, Erlangen, Germany) and a 32‐channel phased array cardiovascular coil by dedicated CMR technologists. The exam included standard breath held segmented cine imaging with a steady‐state free precession (SSFP) sequence. LVM and LVEF were measured from short‐axis stacks of end‐diastolic and end‐systolic cine frames without geometric assumptions. Ten minutes after a 0.2‐mmol/kg intravenous gadoteridol bolus (Prohance; Bracco Diagnostics, Princeton, NJ) LGE imaging was performed. To optimize LGE, a phase‐sensitive inversion recovery (PSIR) pulse sequence was used*,* rendering signal intensity proportional to T1 recovery and corrected for surface coil intensity variation. When patients could not breath hold, single‐shot SSFP, averaged PSIR, motion‐corrected images were acquired.^17^, ^18^ Typical acquisition parameters were field of view 360×270 mm, matrix 256×128 mm, and slice thickness 6 mm.

#### Quantification of the myocardial ECV fraction

ECV measures in myocardium without infarction or nonischemic scar were acquired after the gadolinium bolus, with minimal variation related to time elapsed following the bolus or to heart rate. T1 measures were recorded using an ECG‐gated single‐shot–modified Look Locker inversion recovery sequence. To avoid partial ECV effects, the middle third of the myocardium was the traced region of interest (ROI). A circular ROI was traced in the center of the blood pool as large as possible while avoiding the papillary muscles.

MI was identified when LGE involved the subendocardium in a coronary distribution. Nonischemic scar was defined as not having a distribution of LGE corresponding to MI. We quantified ECV from the myocardium without any apparent scar with the following formula:

ECV=λ·(1−hematocrit)

where λ=ΔR1_myocardium_/ΔR1_bloodpool_ and ΔR1=1/T1_precontrast_−1/T1_postcontrast_. ECV was measured in 2 short‐axis slices in the basal and midventricular level in precontrast and postcontrast T1 images. Postcontrast T1 mapping images were acquired after acquisition of LGE images. These images were usually acquired 20 minutes after the contrast bolus. Hematocrit was acquired on the day of CMR and analyzed in the clinical laboratory. The final ECV measures were averaged from the basal and midventricular short‐axis slices. Because of obliquity between myocardium and the image plane, which could introduce partial volume error, ECV was not measured in apical slices.^19^ CMR data were analyzed using existing clinical software. Furthermore, myocardial extracellular mass and myocardial cellular mass were calculated as LVM×ECV, and LVM×(1−ECV), respectively, and both were indexed to BSA.

### ECG Acquisition and Analysis

ECG data for each subject were collected from the local UPMC‐based ECG system (MUSE Cardiology Information System, Version 8.0 SP2; GE Healthcare, Little Chalfont, UK) and exported into anonymized .xml files with coded subject identification. Data were analyzed digitally using semiautomatic software developed in‐house. The following variables of the ECG were analyzed: Sokolow–Lyon index, defined as the sum of S wave voltage in lead V_1_ (S_V1_) plus the larger of the R wave voltage in lead V5 or V6 (R_V5_ or R_V6_), where LVH is defined as >3.5 mV; Cornell voltage, defined as S_V3_+R_aVL_, where LVH is defined as >2.8 mV for men and >2.0 mV for women; Cornell voltage product, defined as Cornell voltage times QRS duration, where LVH is defined as >244 mV×ms; the 12‐lead QRS voltage sum as the sum of the peak of R to nadir of S in each of the 12 leads; the QRS maximum spatial magnitude, defined as the maximum instantaneous amplitude, in millivolts, of the vectorcardiographic QRS loop^20^ as derived from the 12‐lead ECG^21^; and the QRS duration, defined as the time from the beginning of the Q‐wave to the end of the S‐wave.

### Statistical Analysis

Statistical analysis was performed using SPSS software (version 23; IBM Corp, Armonk, NY). The Kolmogorov–Smirnov test was used to determine whether continuous variables were normally distributed. Differences between the subgroups' baseline data were tested using ANOVA, the Kruskal–Wallis test, or the chi‐square test, as appropriate. Normally distributed measures were described using mean and SD and compared using the Student *t* test. Non‐normally distributed measures were described using median and interquartile range and compared using the Mann–Whitney *U* test. Linear correlations were evaluated using Pearson's correlation coefficient (*r*) and expressed as its square (*R* ^2^). Multivariable linear regression was used to evaluate the relative contributions of ECV, LVMI, EDV, body fat, and LVM/EDV^2/3^ to the investigated ECG variables. Nonsignificant univariable measures were not included in the multivariable analysis. A *P*<0.05 was considered statistically significant.

## Results

Baseline characteristics of the population are presented in Table 1. A total of 77 patients were included in the study: patients with normal ECV and low LVMI (n=20), normal ECV and high LVMI (n=20), increased ECV and low LMVI (n=20), and increased ECV and high LVMI (n=17). Six patients met LVH by Sokolow–Lyon criteria only, 5 by Cornell voltage criteria only, 2 by Cornell product only, and 1 patient by all 3 sets of criteria.

### Subgroup Comparisons

ECG measures for low/high LVMI and normal/increased ECV were compared, respectively. All ECG voltage variables, including Sokolow–Lyon, Cornell, 12‐lead, and QRS maximum spatial magnitude voltages, were higher with greater LVMI and lower with increased ECV (*P*<0.05 for all). However, QRS duration did not differ by either ECV or LVMI.

### Impact of ECV on Voltage/Mass Ratio

**Figure 2:**
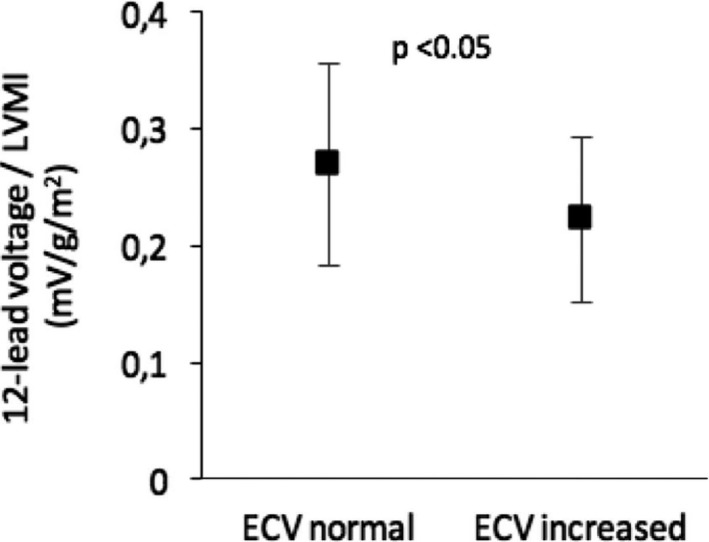
Differences in voltage/mass ratio with regards to extracellular volume fraction (ECV). The voltage mass ratio was measured as the 12‐lead voltage sum divided by left ventricular mass index (LMVI). Data are shown as mean±1 SD.

Figure 2 shows that the voltage/mass ratio, measured as the 12‐lead voltage per LVMI, decreased with increased ECV (*P*<0.05). Thus, each unit of myocardial mass contributed less ECG voltage in the setting of increased ECV.

### Uni‐ and Multivariable Linear Correlations

**Figure 3:**
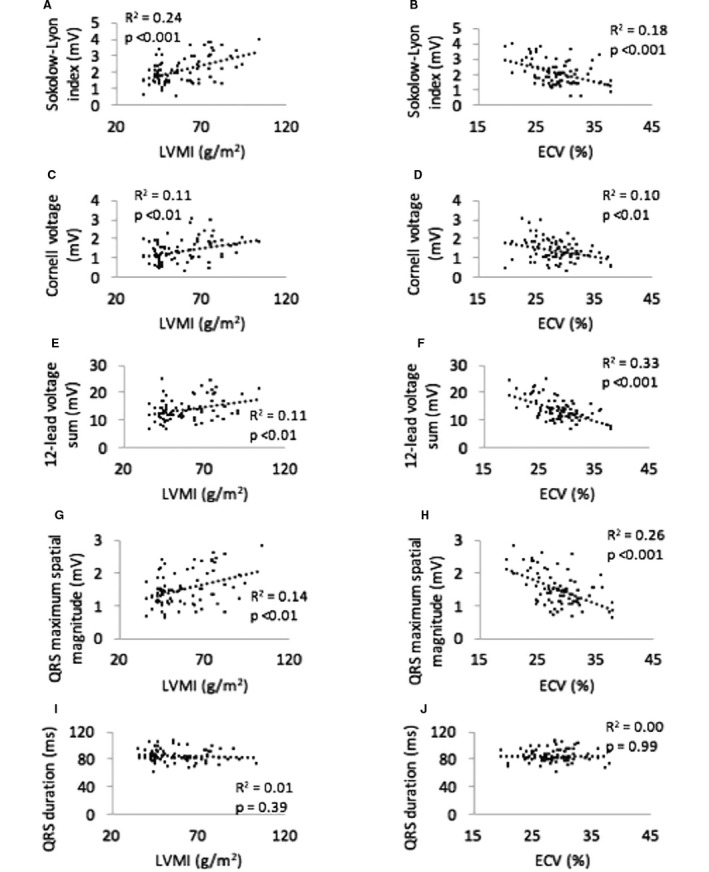
Linear correlations between ECG measures and left ventricular mass index (LVMI) and extracellular volume fraction (ECV). Note that LVMI is positively correlated with ECG voltage, whereas ECV is negatively correlated with ECG voltage. QRS duration is not correlated with LVMI or ECV. Linear correlations between: A, Sokolow‐Lyon Index and LVMI; B, Sokolow‐Lyon Index and ECV; C, Cornell voltage and LVMI; D, Cornell voltage and ECV; E, 12‐lead voltage sum and LVMI; F, 12‐lead voltage sum and ECV; G, QRS maximum spatial magnitude and LVMI; H, QRS maximum spatial magnitude and ECV; I, QRS duration and LVMI and; J, QRS duration and ECV.

As shown in Figure 3, LVMI was positively correlated with all ECG voltage variables whereas ECV was negatively correlated with all ECG voltage variables. QRS duration did not correlate with ECV or LVMI. All correlations remained significant when comparing against LVM rather than LVMI.

**Figure 4:**
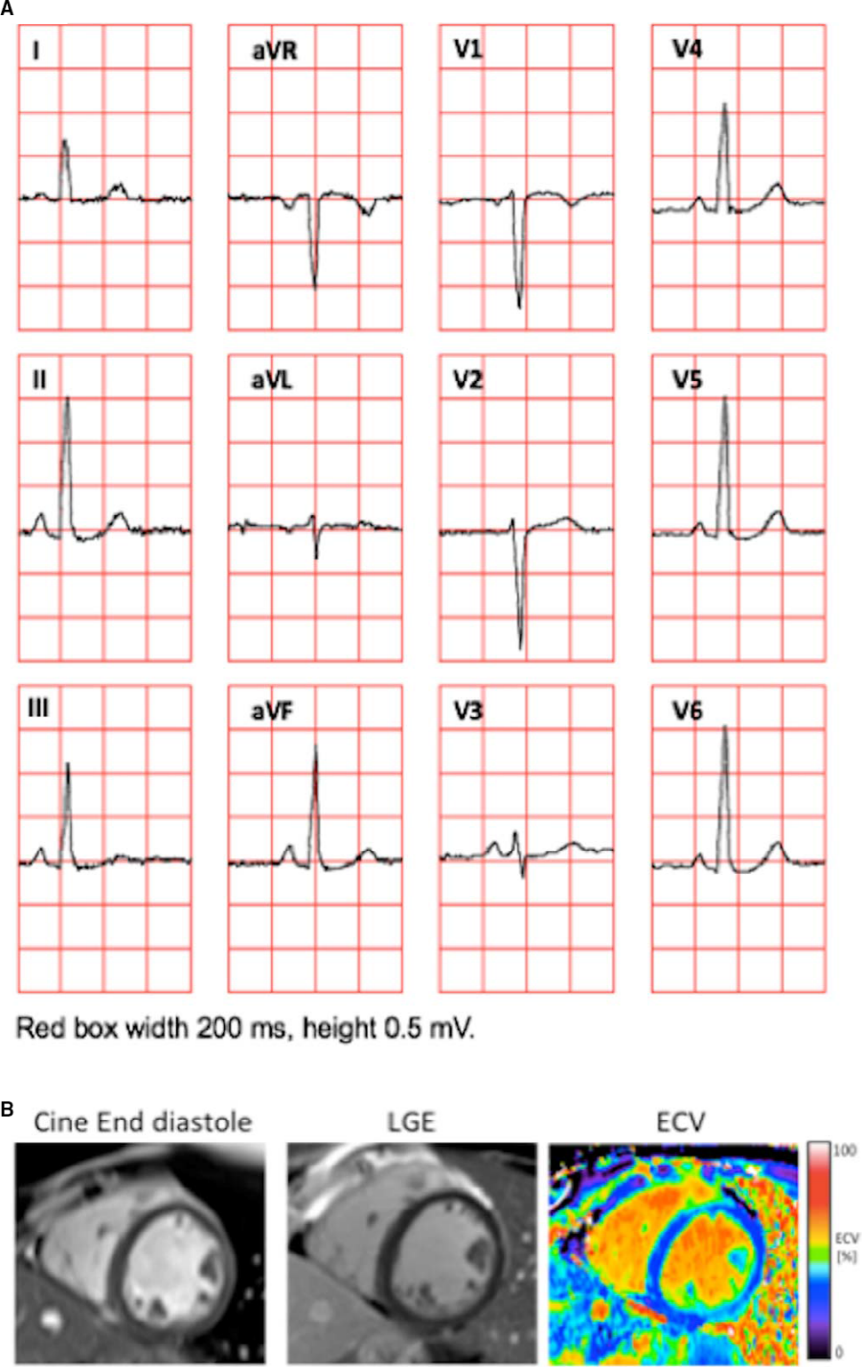
Example of a 24‐year‐old woman illustrating the coexistence of normal QRS amplitudes on the ECG in patients with high left ventricular mass index (LVMI) and increased myocardial extracellular volume fraction (ECV). A, The 12‐lead ECG. The Sokolow‐Lyon index was 2.8 mV, Cornell voltage 0.3 mV, and the 12‐lead voltage was 14.7 mV. B, An end‐diastolic cine image from a midventricular short‐axis slice, which was part of the short‐axis stack from which the LVMI (60 g/m^2^) was measured (left), a corresponding late gadolinium enhancement (LGE) showing absence of focal abnormalities (middle), and a corresponding ECV image showing diffusely increased ECV (30%) consistent with diffuse myocardial fibrosis (right).

Table 2 summarizes the results for uni‐ and multivariable linear regression. The results show that ECV and LVMI contributed approximately equally, but in opposing directions, to the ECG voltage. Both ECV and LVMI were independent predictors of Sokolow–Lyon voltage and QRS maximum spatial magnitude, and together with body fat, EDV, and LVM/EDV^2/3^ they could explain ≈40% of voltage. For the other ECG voltage parameters, ECV was the only independent predictor of voltage. Univariable correlations between Cornell voltage and Cornell product and LVMI and ECV, respectively, were similar in magnitude and significance, as well as the multivariable adjusted *R* ^2^. Figure 4 shows an illustrative case of a subject with high LVMI and ECV, yet absence of increased voltage amplitudes on the ECG. Furthermore, ECG voltage correlations with myocardial extracellular mass index and myocardial cellular mass index were also explored, but did not yield independent incremental information (data not shown). ECV and LVMI were not correlated with each other (*R* ^2^=0.02; *P*=0.25). There was a weak correlation between LVEF and LVMI (*R* ^2^=0.05; *P*=0.04), but there was no correlation between LVEF and ECV (*R* ^2^=0.01; *P*=0.52).

**Table 2:** Uni‐ and Multivariable Linear Regression Between ECG Variables and LVMI, ECV, Body Fat, EDV, and LVM/EDV^2/3^, Respectively

ECG Variable	Univariable *r*, LVMI (*P* Value)	Univariable *r*, ECV (*P* Value)	Univariable *r*, Body Fat (*P* Value)	Univariable *r*, EDV (*P* Value)	Univariable *r*, LVM/EDV^2/3^ (*P* Value)	Multivariable β, LVMI (*P* Value)	Multivariable β, ECV (*P* Value)	Multivariable β, Body Fat (*P* Value)	Multivariable β, EDV (*P* Value)	Multivariable β, LVM/EDV^2/3^ (*P* Value)	Multivariable Adjusted *R* ^2^ (*P* Value)
Sokolow–Lyon index, mV	0.49 (<0.001)	−0.43 (<0.001)	−0.27 (0.010)	0.37 (<0.001)	0.37 (0.001)	0.51 (0.045)	−0.34 (0.001)	0.16 (0.10)	0.05 (0.70)	−0.11 (0.62)	0.42 (<0.001)
Cornell voltage, mV	0.34 (0.001)	−0.32 (0.002)	−0.006 (0.48)	0.18 (0.06)	0.31 (0.003)	0.25 (0.21)	−0.28 (0.011)	N/A	N/A	0.06 (0.76)	0.19 (0.001)
Cornell product, mV×ms	0.28 (0.006)	−0.29 (0.006)	−0.034 (0.38)	0.23 (0.023)	0.27 (0.008)	−0.16 (0.60)	−0.22 (0.055)	N/A	0.25 (0.13)	0.36 (0.18)	0.17 (0.008)
12‐lead voltage, mV	0.33 (0.002)	−0.57 (<0.001)	−0.24 (0.019)	0.21 (0.034)	0.27 (0.009)	0.46 (0.07)	−0.53 (<0.001)	−0.13 (0.16)	−0.10 (0.47)	0.19 (0.40)	0.42 (<0.001)
QRS maximum spatial magnitude, mV	0.38 (0.001)	−0.51 (<0.001)	−0.26 (0.013)	−0.27 (0.008)	0.27 (0.010)	0.58 (0.03)	−0.46 (<0.001)	−0.15 (0.12)	−0.077 (0.60)	−0.27 (0.23)	0.39 (<0.001)
QRS duration, ms	−0.10 (0.20)	0.00 (0.50)	−0.13 (0.12)	0.24 (0.017)	−0.07 (0.28)	N/A	N/A	N/A	N/A	N/A	N/A

Nonsignificant univariable measures were not included in the multivariable analysis. ECV indicates extracellular volume; EDV, end‐diastolic volume; LVM, left ventricular mass; LVMI, left ventricular mass index; N/A, not applicable.

## Discussion

The major finding of this study is that there is a positive relationship between ECG voltage amplitude and LVMI as well as an inverse relationship between ECG voltage amplitude and ECV. ECV and LVMI were not related to each other. After excluding confounding conditions, and when accounting for body fat, EDV, and LVM/EDV^2/3^, a surrogate for mass‐to‐volume ratio, we found that both LVMI and ECV were sole independent predictors of QRS voltage.

To our knowledge, this is the first investigation to show the influence of diffuse myocardial fibrosis upon ECG criteria for LVH. A plausible physiological explanation for these findings is that increased LVM may give rise to larger and longer living activation boundaries, which thereby increase QRS amplitude. However, the complex process beyond the increased LVM, such as myocyte hypertrophy and apoptosis, changes in contractile and electrical phenotype, and alterations in the quantity and composition of the extracellular matrix, are additional factors that have been shown to affect QRS voltage.^22^ Conversely, increased ECV, consisting mostly of collagen, might serve as an electrical insulator, thus decreasing the voltage recorded by ECG, a phenomenon also observed in cardiac amyloidosis whereby extensive interstitial protein deposition occurs.^23^ Our results indicate that increased ECV may disguise the ECG manifestations of increased LVM, in line with the previously described concept of “relative voltage deficiency.”^24^

ECV and LVMI did not correlate with each other, and this has previously been postulated by others.^3^ There is some evidence indirectly supporting the notion that increased ECV is associated with decreased ECG voltages. For instance, the increase in both LVM and ECV associated with cardiac amyloidosis has been shown to correlate with decreased ECG voltages.^25^, ^26^ Furthermore, both focal and diffuse myocardial fibrosis have been suggested to affect the impulse propagation recorded by ECG through at least 3 distinct mechanisms: decreases in the proportion of electrically active tissue; slowing of impulse generation; and fractionation of the electrical front.^27^, ^28^

We found no relationship between LVMI (or LVM) and QRS duration, nor a relationship between QRS duration and ECV. Previous studies have found a relationship between QRS duration and LVM measured by echocardiography or CMR.^29^, ^30^, ^31^, ^32^, ^33^ It may be that relationships between LVM and QRS duration are more pronounced in the high ranges of both variables. Thus, the reason for the discrepancy may lie in our strict selection of subjects to avoid confounders, given that all patients in our study had a QRS duration <110 ms. Notably, however, in the current study, both LVMI and ECV correlated with voltage measures regardless of the absence of correlation with QRS duration. ECV, but not LVMI, was an independent predictor for Cornell voltage and 12‐lead voltage. LVM/EDV^2/3^, was correlated with all ECG voltage variables; however, it was not an independent predictor for ECG voltage. However, mean LVM was lower in the current study compared to a previous study,^12^ resulting in relatively low LVM/EDV^2/3^ values. It may be that LVM/EDV^2/3^ may predict ECG voltage in the higher ranges of LVM/EDV^2/3^.

The ECG has repeatedly been shown to lack both sensitivity and specificity in diagnosing LVH.^34^, ^35^ The Journal of Electrocardiology LVH Working Group has proposed that instead of the currently widely accepted model, that is, that heart disease leads to increased myocardial mass that causes ECG abnormalities, it is likely that heart and vascular disease lead to structural, bioelectrical, and biochemical changes that cause ECG abnormalities.^36^ The results of the current study add knowledge to this ongoing debate, including the discussion related to the use of the term left ventricular electrical remodeling (LVER) as opposed to “ECG‐LVH.”

### Study Limitations

Because of the characteristics of the subjects available for the study, the cohort was grouped according to high/low LVMI based on values above or below the median of the population as opposed to above or below the upper limit of normal values, which may vary by technique. However, we judged this grouping strategy suitable for studying the basic relationships between ECG, ECV, and LVMI, respectively. A further limitation was that neither age nor sex was taken into account when exploring these relationships. Stratifying the population according to age and sex would require increased sample size to maintain adequate power, and such studies are justified. Notably, age and sex distribution did not differ across the 4 subgroups of high/low LVMI and normal/increased ECV. The current study did not address the effects of focal myocardial scarring attributable to infarction or nonischemic scar upon the ECG, and thus the current findings may not be applicable to such a population. However, the current study was specifically designed to enroll a carefully selected population without myocardial scar in order to specifically address the isolated and respective effects of diffuse myocardial fibrosis and LVM on ECG. We believe that such a carefully selected study population was an important and unique strength of the study necessary to address the hypothesis.

### Clinical Applications

The present study provides the first in vivo data supporting a novel mechanistic explanation behind the lack of sensitivity of voltage‐based ECG criteria for detecting increased LVM. Also, the results illustrate that some patients may have pathologically increased ECV that can normalize voltages and thereby disguise ECG detection of LVH.

## Conclusions

Myocardial mass and diffuse fibrosis have independent and opposing effects upon ECG voltage measures of LVH. Thus, diffuse myocardial fibrosis may disguise the ECG manifestations of increased LVM. These competing effects reflect LV structural and electrical remodeling of the hypertrophied myocardium and provide mechanistic insight into the limited sensitivity of the ECG for detecting increased LVM.

## Sources of Funding

The study was funded, in part, by the Swedish Research Council, Swedish Heart and Lung Foundation, Stockholm County Council, and Karolinska Institutet. This work was supported by a grant from The Pittsburgh Foundation (PA), Grant M2009‐0068; and an American Heart Association Scientist Development grant (09SDG2180083) including a T. Franklin Williams Scholarship Award; funding provided by: Atlantic Philanthropies, Inc., the John A. Hartford Foundation, the Association of Specialty Professors, and the American Heart Association (Dallas, TX). This work was also supported by Grant No. UL1 RR024153 from the National Center for Research Resources (NCRR), a component of the National Institutes of Health (NIH), and NIH Roadmap for Medical Research.

## Disclosures

Dr Schlegel reports ownership interest; Modest; Nicollier‐Schlegel Sarl. Dr Schelbert has accepted contrast material from Bracco Diagnostics for research purposes beyond the scope of this work. Dr Wong is supported by a grant from the American Heart Association and the Children's Cardiomyopathy Foundation. Dr Ugander is principal investigator for the Karolinska Cardiovascular Magnetic Resonance Group, and Karolinska University Hospital has a collaborative research and development agreement with Siemens regarding cardiovascular magnetic resonance imaging.
